# Effects of rabbit pinna-derived blastema cells on tendon healing 

**DOI:** 10.22038/IJBMS.2019.29102.7045

**Published:** 2020-01

**Authors:** Nooshin Ghayemi, Farshid Sarrafzadeh-Rezaei, Hassan Malekinejad, Mehdi Behfar, Amir-Abbas Farshid

**Affiliations:** 1 Department of Surgery and Diagnostic Imaging, Faculty of Veterinary Medicine, Urmia University, Urmia, Iran; 2 Department of Pharmacy and Toxicology, Faculty of Pharmacy, Urmia University of Medical Sciences, Urmia, Iran; 3 Department of Pathology, Faculty of Veterinary Medicine, Urmia University, Urmia, Iran

**Keywords:** Cell- and tissue-based – therapy, Collagen, Ear auricle, Hydroxyproline, Regenerative medicine, Tendons

## Abstract

**Objective(s):**

Tendon healing is substantially slow and often associated with suboptimal repair. Cell therapy is one of the promising methods to improve tendon repair. Blastema, a population of undifferentiated cells, represents characteristics of pluripotent mesenchymal stem cells and has the potentials to be used in regenerative medicine. The aim of this study was to investigate the use of blastema allotransplantation in rabbit tendon healing.

**Materials and Methods:**

In this study, one rabbit was used as a blastema donor, and twenty-four rabbits were divided into control and treatment groups. Blastema cells were obtained from ear pinna upon punch hole injury in the donor rabbit. Under general anesthesia, a complete transverse tenotomy was performed on the midsubstance of deep digital flexor tendon followed by suture-repair. In the treatment group, 1 × 10^6^ blastema cells suspended in buffer saline were injected intratendinously at the repair site, while the control group received only the buffer saline. Cast coaptation was maintained for two weeks. Eight weeks after the operation, tendons were harvested, and histopathological, biomechanical, and biochemical assays were performed on samples.

**Results:**

Mechanical testing showed a significant increase in ultimate load, energy absorption, stiffness, yield load, stress, and strain in blastema-treated tendons compared to controls. Also, higher hydroxyproline content and improved collagen alignment along with lower inflammatory cell infiltration and decreased angiogenesis were observed in blastema-treated tendons.

**Conclusion:**

Increased levels of hydroxyproline and improved histopathological and biomechanical parameters in the treatment group suggest that blastema cells could be considered an adjunct to tendon repair in rabbits.

## Introduction

One of the most common orthopedic problems is tendon injury being difficult to manage (1). Tendons have minimal regenerative capacity compared to other tissues due to poor vascularization and low cellularity (2). Tendon healing is associated with fibro-vascular scar tissue formation having a negative effect on rehabilitation and normal functionality (1-3). Therefore, treatment of tendon injuries is still a great challenge for orthopedic surgeons (4). In tendon healing, the gold standard technique is surgical repair of the tendon via primary suture or autologous transfer. However, still there remain some challenges in these techniques, such as reduced mechanical strength of cured tendon due to scar tissue formation, infection and donor site morbidity as well as limited availability of autografts (5). Due to the inconsistency in the results of traditional methods, alternative treatments like cell therapy have been designed to provide optimal healing (3). The utilized cell sources in ligament repair have included the cells originating from the intact ligament, such as tenoblasts and tenocytes, and also the stem cells emanated from different origins such as bone marrow or adipose tissue (6).

Although there is a potential for the above-mentioned cell types’ application, more suitable resources of progenitors have always been sought (2). Blastema, a cellular mass forming by a group of undifferentiated cells capable of differentiating into the lost tissues during the regeneration process, could be regarded as a new tissue banking model (7).

Regeneration, the ability to recreate lost or damaged tissue, organs, and limbs, is mostly seen in lower vertebrates, especially amphibians. Nevertheless, a few mammals have also shown this ability (8, 9). The regeneration capability has also been documented in the human embryo, where the damaged embryo is born without a scar as the embryonic stem cells heal the defect through blastema mechanism (10). The adult human body usually has different responses to injured tissue tending to form dermal scars in healing wounds, and the relative inadequacy of regeneration may be attributed to this scar formation precluding blastema production (11, 12). Digit tip is the only part of the human body preserving the regenerative capabilities (12, 13).

Rabbit pinna possesses a unique regenerative capability after punching in which all of the lost structures can be restored. This kind of ear scar-free regeneration is achieved by blastema formation and subsequent cartilage regrowth and hair follicle and sebaceous gland reconstruction (14-16). Blastema contains unique, powerful progenitor cells with high proliferative capability being influenced by environmental signals (17).

According to the previous studies, it has been suggested that blastema from rabbit ear consists of a group of undifferentiated cells capable of differentiation into bone, cartilage, and adipose cells with the tendency of *in vitro *clonogenic growth resembling mesenchymal stem cells (MSCs). Blastema cells have also shown expression of genes such as OCT4 and SOX2, a characteristic of embryonic stem cell (18-22). 

The present study aimed to investigate the effects of blastema cells from rabbit pinna on the repair of the surgical model of acute tendon injury in rabbits. It was hypothesized that the application of stem-like cells of blastema would improve histopathological, biomechanical, and biochemical properties of experimentally induced tendon injury. 

## Materials and Methods

All stages of this research were performed according to the guidelines of the Ethics Committee, and Urmia University Research Council approved all experiments (3/T-TD/1810). In the present study, 25 healthy adult male New Zealand White (NZW) rabbits (*Oryctolagus cuniculus*), weighing approximately 2.50–3.00 kg were used. The animals were kept under standard conditions and given commercial rabbit pellet (Urom-Dordaneh Feed Mill, Urmia, Iran) and tap water. One rabbit was used as a blastema donor, while the other twenty-four rabbits were divided randomly and equally into control and treatment groups, each having twelve NZW rabbits.

***Preparation and culture of blastema***

The donor rabbit was sedated by intramuscular injection of ketamine (25.00 mg kg^-1^; Alfasan, Woerden, The Netherlands) and xylazine (5.00 mg kg^-1^; Alfasan, Woerden, The Netherlands) and one ear pinna was prepared aseptically. In order to create a 2 mm diameter hole, the first punch of pinna was performed with a tissue puncher (Biopunch®, Ted Pella, Inc., USA) between medial ear artery and marginal ear vein. A few days after punching of the first hole, the blastema ring was formed in the periphery of the primary hole. Four days later, the second punch (4 mm in diameter) was performed on the primary hole, and an O-shaped ring was obtained. To obtain a single-cell suspension, the small pieces from the internal part of O-shaped ring following cleaning and washing twice with phosphate buffer saline (PBS; pH: 7.20) and culture medium, were treated with 0.40% collagenase/Dulbecco’s modified Eagle’s medium (DMEM; Gibco, Darmstadt, Germany) for 30 min at 37 ^°^C. To stop the reaction, an equal volume of culture medium containing 10% fetal bovine serum (FBS; Gibco, Darmstadt, Germany) was added. After that, tissue suspension was passed through a 70 µm cell strainer and then centrifuged at 500 g for 5 min at 4 ^°^C. The pellet was re-suspended by pipetting, counted through trypan blue staining technique, cultured in a cell culture flask (T25) containing DMEM supplemented with 10% FBS and 100 IU ml^-1^ penicillins and 100 μg ml^-1^ streptomycin (Sigma-Aldrich, Germany) and incubated in an atmosphere of 37 ^°^C and 5% CO_2_. During one-week incubation, the cells started to adhere to the surface of cell culture flask and on days 9–12, the first subculture was performed. After four passages, most of the cells were found fully adherent with the fibroblast-like phenotype (23).

***Surgical procedure***

The NZW rabbits were anesthetized by intramuscular injection of 40.00 mg kg^-1^ ketamine and 5.00 mg kg^-1^ xylazine. One hind limb from each rabbit was randomly prepared for surgical operation. 

**Figure 1:**
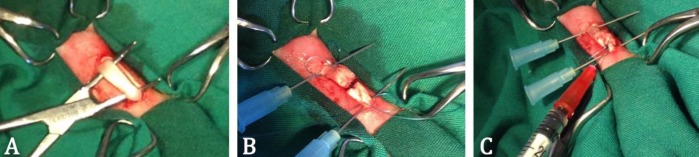
Intraoperative photographs exhibiting A) Exposed deep digital flexor tendon; B) Modified Kessler suture placement after complete transection of tendon; and C) Intratendinous injection of blastema at the repair site

Aseptically, a longitudinal skin incision was made on the plantar surface of metatarsus, and the deep digital flexor tendon (DDFT) was exposed through dissection. For experimental tenotomy, a sharp complete transection was performed through midsubstance of DDFT. The transected tendon ends were immediately sutured with 3/0 monofilament nylon (Supa Medical Devices Co., Tehran, Iran) in a modified Kessler pattern. In the treatment group, 0.10 ml PBS solution containing 1×10^6^ blastema cells was injected intratendinously (5) at both proximal and distal tendon stumps just next to the transection line. The same surgical procedure was performed on the rabbits in the control group except that they only received equal volume of PBS solution for intratendinous injection (Figure 1). The skin incision was closed by cross mattress using 3/0 monofilament nylon suture. The operated limbs were immobilized using below-stifle fiberglass cast (Tehran Ortho Co, Tehran, Iran) for two weeks after the operation.

Rabbits were euthanized by intravenous injection of thiopental sodium overdose (50.00 mg kg^-1^, Sandoz, GmbH, Kundl, Austria) eight weeks after surgery. For biomechanical tests, eight rabbits were selected randomly from each group, and then tendons from both hind limbs were harvested. All samples were wrapped in normal saline soaked sterile gauzes and frozen at −20 ^°^C until the tensile test. 

Tendons from the remaining four rabbits in each group were evenly divided longitudinally into two halves, and each half was subjected to either biochemical assay or histopathological examination. Also, four intact tendons from the unoperated contralateral hind limbs were randomly harvested to measure hydroxyproline concentration. In this regard, samples were placed in aluminum foil, frozen by exposure to liquid nitrogen, and then kept at −20 °C. For histopathological examinations, tendon specimens were fixed by immersion in 10.00% neutral-buffered solution immediately after sampling.

***Biomechanical study***

Prior to performing tensile testing, the frozen DDFT samples were kept at room temperature for two hours and allowed to thaw. Suture material was also removed from all samples. All tests were conducted at room temperature (24).

The biomechanical evaluations were performed using a universal testing machine (model STM-20; Santam, Tehran, Iran) equipped with a load cell of 500 N. To prevent specimens slipping from the jaws during tensile testing, 360 grit sandpaper was wrapped on either end of each specimen. The distance between the clamping jaws was initially set to 30 mm. Dynamic testing took place under axial tension with a constant speed of 50 mm min^-1^ (6, 24, 25). The biomechanical testing consisted of a single load to failure cycle. The force and elongation of the tendon were recorded constantly by a computer until tendon failure. The force-elongation curve was plotted for each test, and biomechanical parameters including ultimate load (N), yield load (N), energy absorption (J), stiffness (N mm^-1^), stress (MPa), and strain (%) were obtained. These data were provided by STM-controller software (Santam, Tehran, Iran). 

***Histopathological examinations***

Longitudinal sections (5 μm in thickness) were stained with hematoxylin and eosin and evaluated under a light microscope (Nikon, Tokyo, Japan). Parameters, including inflammatory cell infiltration, angiogenesis (vascularization), collagen alignment, and fibroblast distribution, were analyzed by a pathologist in a blind manner. To analyze the fibroblast distribution, angiogenesis, and inflammatory cell infiltration, the cell and vessel numbers per ¼ mm^2^ of tissue were counted and compared between groups. The collagen tangential alignment, as well as collagen expansion and condensation, were analyzed in 2500×2500 µm of tissue using Image-Pro Insight software (Media Cybernetic, USA, Version 9:00). The collagen expansion and condensation were presented by 3-D surface plot graphs. The images were resized and merged by Adobe Photoshop CC 2018 (Version 19.1). 

***Hydroxyproline analysis***

In order to determine hydroxyproline concentration, the intact and operated tendons were homogenized using KCl (150 mM, pH 7.40). 0.50 ml of homogenate was digested in 1.00 ml of 6 N HCl for 8 hr at 120^ °^C. Then, to oxidize the free hydroxyproline, 50 μl of citrate/acetate buffer (5.00% citric acid, 7.24% sodium acetate, 3.40% sodium hydroxide, and 1.20% glacial acetic acid, pH 6.00) and 1 ml of chloramine-T solution (282 mg of chloramine-T, 2.00 ml of n-propanol, 2.00 ml of H_2_O and 16 ml of citrate/acetate buffer) were added to 50 μl of samples and kept at room temperature for 20 min. In the next step, 1.00 ml of Ehrlich’s solution (2.50 g of 4-dimethylamino benzaldehyde, 9.30 ml of n-propanol, and 3.90 ml of 70.00% perchloric acid) was added to each sample, and then the samples were placed in a water bath at 65 ^°^C for 15 min. After cooling down, the sample absorbance was measured at 550 nm (Stat Fax® 2100; Awareness Technology Inc., Palm City, USA). A concentration range of 0.00 to 10.00 μg ml^-1 ^hydroxyproline standard was used to establish a standard curve (26). All chemicals used for this analysis were purchased from Sigma-Aldrich (Germany).

***Statistical analysis***

The student *t*-test was used for quantitative histopathological parameters. The hydroxyproline contents and mechanical properties of tendons were analyzed by one-way ANOVA, followed by Tukey’s *post hoc* test. All statistical analyses were performed using Statistical Package for Social Sciences (SPSS) software (version 21, IBM Corp., Armonk, USA), and *P*-values less than 0.05 were considered to be statistically significant. Data represented as mean±SD.

## Results

**Figure 2:**
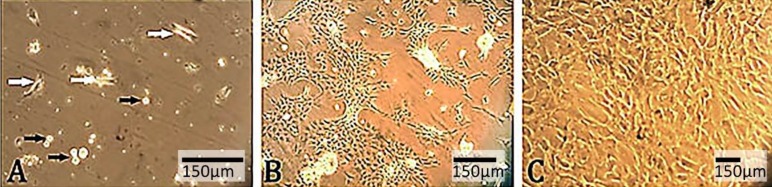
The culture of blastema. A) The primary culture of blastema includes fibroblastic cells (white arrows) and flattened epithelial cells (black arrows); B) Day eighth; and C) Day fourteenth of cell culture when cultures have become homogenous after several subcultures having blastema

The technique for blastema stem cell isolation resulted in yielding of 75.00% alive cells, in which after four passages, adherent cells with fibroblast-like phenotype were observed (Figure 2). 

**Figure 3:**
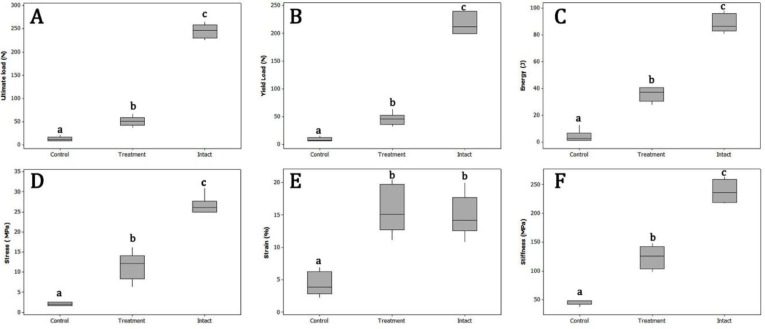
The results of the ultimate load (A), yield load (B), absorbed energy (C), maximum stress (D), strain (E), and stiffness (F) of the samples. abc Different letters indicate significant differences (*P*<0.05) among the groups in each panel

The results of the biomechanical study are illustrated in Figure 3. The mean values of the ultimate load, yield load, and stiffness of the treatment samples were respectively 51.13±10.27 N, 46.13±10.48 N, and 124.40±19.62 MPa, which were found significantly (*P*=0.000) higher than those of the control group (13.44±4.92 N, 9.81±3.62 N and 47.95±7.23 MPa, respectively).

The mechanical study also revealed that the blastema-treated tendons considerably absorbed more energy compared to control ones until failure (*P*=0.000). The absorbed energy of the treatment and control samples was 36.07±5.20 J and 4.40 ± 3.99 J, respectively. In addition, the results indicated that the maximum stress value of the treatment group (11.40 ± 3.40 MPa) was significantly (*P*=0.002) higher than that of the control (2.25±0.82 MPa) group. Regarding strain, the results revealed that the treatment samples had significant (*P*=0.000) higher strain value in comparison with control ones (16.23±3.55% versus 4.27±1.80%, respectively). 

**Figure 4:**
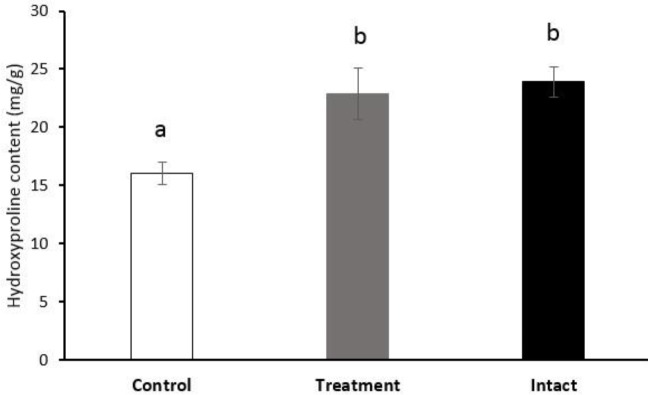
Hydroxyproline content of control and blastema-treated groups and intact tendons

The results of hydroxyproline analysis in the intact tendon (23.90±1.29), control (16.04±0.94), and blastema-treated (22.84±2.22) samples showed that the amount of hydroxyproline was significantly (*P*=0.001) higher in the blastema-treated tendons compared to control ones (Figure 4). No significant difference was observed regarding the hydroxyproline contents between blastema-treated and intact tendons (*P*=0.632).

**Figure 5:**
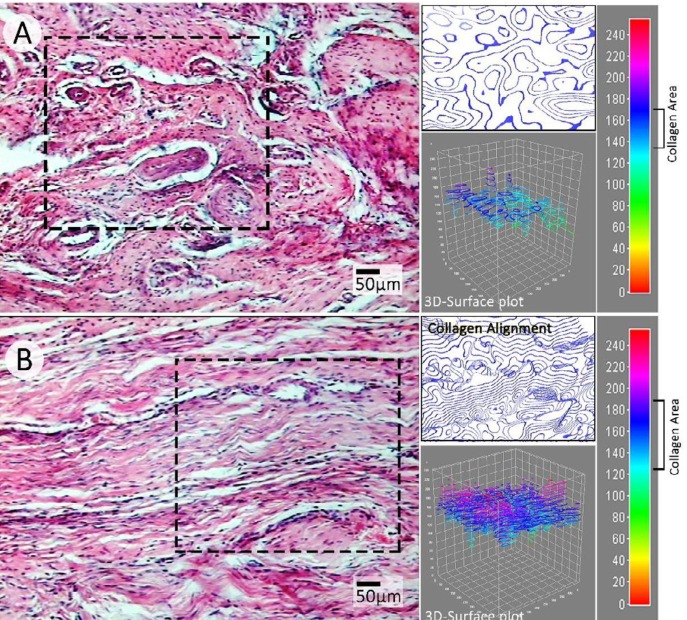
Histological sections of control (A) and blastema-treated (B) groups ( hematoxylin and eosin staining). Note the collagen alignment representing an irregular orientation in the control group. Meanwhile, the software analysis of the section from the blastema-treated group represents a regular alignment. The 3D-surface plot of sections from the treatment group (panel B) represents expanded and condensed collagen in 2500x2500 µm of tissue (graphs are from marked area in hematoxylin and eosin-stained sections) versus control (panel A) group

**Figure 6:**
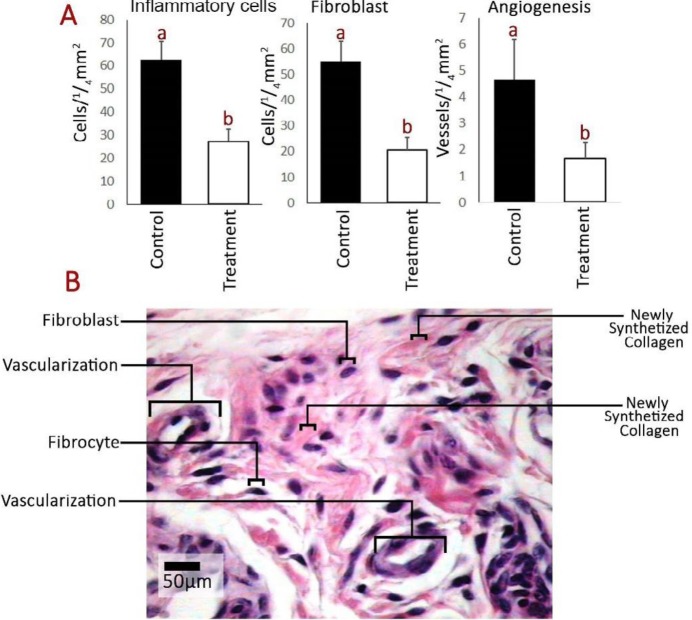
A) Inflammatory cell, fibroblast, and vessel number per ¼ mm^2^ of tissue in different groups. ab Different letters represent significant differences between groups (*P*<0.05). B) Photomicrograph of the healing process (hematoxylin and eosin staining)

Histopathological examinations revealed that the blastema-received animals illustrated tendons with regular collagen alignment (Figure 5). Moreover, to find out the improvement ratio, collagen expansion and condensation were analyzed using software analysis. Accordingly, the cross-sections from blastema-received animals represented improved collagen expansion and condensation versus the control group (note 3-D surface plot graphs in Figure 5). In this line, software analysis exhibited an expanded collagen area as well as condensed collagen alignment in blastema-received group cross-sections (2500×2500 µm). Further, the blastema-received group represented lower vessel number per ¼ mm^2^ of tissue compared to the control group. Additionally, the fibroblast distribution was analyzed and compared between groups. The blastema-received group represented reduced fibroblast distribution per ¼ mm^2^ versus the control group (Figure 6). 

## Discussion

This study showed that local transplantation of blastema cells has the potential to promote mechanical, biochemical, and histopathological properties of neotendons in a rabbit model of DDFT repair.

Cell therapy is one of the promising options to support tendon and ligament healing and improves the biomechanical properties of the regenerated tissues (6, 27). In this regard, the regenerative potency of stem cells from different origins such as bone marrow (25, 28), adipose tissue (29), stromal vascular fraction (24), circulating stem cell (30), and fibroblast-like synoviocytes (31) has been investigated in tendon healing. 

There are several ways of applying cells to promote the healing process. Availability and effectiveness are two important factors having an impression on the surgeon’s interest in a particular application method in research and clinics. Nonetheless, it has always been taken into account that some procedures are more time-consuming and costly than others, for instance, decellularization of natural tissues and reseeding them by cells compared to merely injecting the corresponding cells to the site of injury (6). In the present study, we chose single intratendinous injection of blastema cells at the site of lesion to focus on the effects of blastema cells on tendon healing.

The biomechanical test has been known as the gold standard for efficacy assessment of any method in tendon regeneration or repair (32). In the present study, the results of the mechanical evaluations demonstrated that tensile strength parameters, including ultimate load, energy absorption, stiffness, yield load, stress, and strain were significantly higher in blastema-injected tendons compared to control ones receiving no cells. A rise in the ultimate and yield loads observed in the treatment group indicates that these neotendons can withstand higher tensile loads compared to placebo-received tendons (33). Previous reports have demonstrated that increased stress value is a sign of improvement in the specimens’ quality due to the biochemical characteristics and matrix quality improvement (34). In this study, collagen fibers deposition and organization increase can be possibly the cause for higher stress observed in the treatment group. Furthermore, to reserve and release the high loads that tendon bears during daily activities without imposing any damage to its tissue, a great energy-absorbing capacity is required. Otherwise, an increased risk of tendon overload and re-injury may result. Thus, tendon energy capacity improvement should be taken as one of the key points in tendon injury treatments (35, 36). In this study, the significant increase in energy absorption capacity in the treatment group could be attributed to the reparative effects of treatment with blastema cells.

Tendon stiffness and strain are dependent on the quality of collagen cross-links (37). In fact, collagen cross-links are involved in the elastic and viscoelastic properties of tendons, and ligaments encounter the tensile forces (38). Reportedly, the maturation phase, which is associated with increased cross-linking of collagen fibers begins around ten weeks after injury. The cross-links are responsible for tissue stiffness (39), and it seems that in the current study, blastema cell transplantation accelerated the commencement of maturation phase eight weeks after surgery, as a higher stiffness was observed in the treatment group. The non-significant difference in strain between blastema-treated tendons and intact ones was a surprising **‎**result that may have two explanations: I) as a result of regenerative properties of blastema cells, the visco-elastic characteristics of neotendons **‎**have been improved **‎** ‎and (II) the potential error arising from the slight slipping from the clamps during the **‎**tensile test of intact tendons has led to extended displacement of upper clamp and increased strain value to 14.862±3.05%. The latter has been reported as a common problem during tensile test performance (36, 40).

The results of the current study were in agreement with Behfar *et al*. (24) and Javanmardi *et al*. (25) findings respectively recruiting adipose-derived stromal vascular fraction and bone marrow-derived mesenchymal stem cells to help tendon healing.

The tensile strength of tendons is co-related to the collagen content, type of collagen fibers, and the quality of collagen fiber cross-links (41, 42). There is a lower proportion of type I collagen and an increase in the amount of type III collagen in torn tendons. Type III collagen has a lower level of cross-linking compared to type I; therefore, the mechanical strength will decrease (43). The high biomechanical properties observed in blastema transplanted tendons possibly resulted from acceleration and improvement of the remodeling phase relating to the increased collagen turnover and cross-linking and enhanced granulation tissue organization; since the better remodeling phase occurs, the more repair site strength appears (44). Our histopathological findings support the improved biomechanical properties as the collagen alignment on the one hand and remarkably reduced angiogenesis, on the other hand, were substantially noted in blastema-received tendons.

One of the main amino-acids found in collagen molecules is hydroxyproline, and its quantification is used as an indirect quantification of tissue total collagen content (45). In the present study, blastema improved the hydroxyproline concentration compared to the controls, which means a significant increase in collagen fibers synthesis resulting in improved biomechanical functionality and collagen alignment. 

The pluripotency and great self-renewal capacity of blastema cells provide a suitable source for regeneration studies (7, 18-20). Furthermore, this cell mass could be influenced by environmental signals and consequently transformed into different cell types (18). Predominant proliferative and clonogenic properties compared to MSCs, being immortal cells, and adaptive to *in vitro* culture condition, having a higher level of lineage-specific genes, and capability of differentiation are considered as other characters of blastema cells making them a valuable and potential therapeutic source in regenerative medicine (18-22, 46-48). Moreover, the capability of blastema cells to migrate into decellularized three-dimensional matrix of ovine bladder and to differentiate into epitheliocytes, fibroblasts, and adipocytes has been reported formerly (49). 

ab Different letters indicate significant differences among groups (*P*< 0.05)

## Conclusion

The present study showed that the transplantation of blastema cells in experimentally-injured tendon resulted in improved healing of neotendons, as evidenced by biomechanical, histopathological, and hydroxyproline content analyses. These findings support the efficacy of blastema in the treatment of tendon injuries. However, further studies are recommended to disclose blastema cell induced ultra-structural alterations. 

## References

[1] Galatz LM, Gerstenfeld L, Heber-Katz E, Rodeo SA (2015). Tendon regeneration and scar formation: the concept of scarless healing. J Orthop Res.

[2] Longo UG, Lamberti A, Maffulli N, Denaro V (2010). Tissue engineered biological augmentation for tendon healing: a systematic review. Br Med Bull.

[3] Hampson K, Forsyth N, El Haj A, Maffulli N (2008). Tendon tissue engineering. Top Tissue Eng.

[4] Koob TJ (2002). Biomimetic approaches to tendon repair. Comp Biochem Physiol A.

[5] Alshomer F, Chaves C, Kalaskar DM (2018). Advances in tendon and ligament tissue engineering: materials perspective. J Mater.

[6] Buschmann J, Meier Bürgisser G (2017). Biomechanics of tendons and ligaments.

[7] Mahdavi Shahri N, Naseri F, Kheirabadi M, Babaie S, Azarniya M (2008). The ultra-structural study of blastema in pinna tissues of rabbits with transmission electron microscope. J Biol Sci.

[8] Tsonis PA (2002). Regenerative biology: the emerging field of tissue repair and restoration. Differentiation.

[9] Brockes JP, Kumar A (2002). Plasticity and reprogramming of differentiated cells in amphibian regeneration. Nature Rev Mol Cell Biol.

[10] Muneoka K, Han M, Gardiner DM (2008). Regriowing human limbs. Sci Am.

[11] Simkin J, Seifert AW (2018). Concise review: translating regenerative biology into clinically relevant therapies: are we on the right path?. Stem Cells Transl Med.

[12] Dunk SA (2012). Limb regeneration in humans: micromanaging a plastic environment. Vanderbilt Undergrad Res J.

[13] Simkin J, Sammarco MC, Dawson LA, Schanes PP, Yu L, Muneoka K (2015). The mammalian blastema: regeneration at our fingertips. Regeneration.

[14] Goss RJ, Grimes LN (1975). Epidermal downgrowths in regenerating rabbit ear holes. J Morphol.

[15] Grimes LN, Goss RJ (1970). Regeneration of holes in rabbit ears. Am Zool.

[16] Williams-Boyce PK, Daniel JC (1980). Regeneration of rabbit ear tissue. J Exp Zool.

[17] Alizadeh M, Mahdavi N, Rezaeeian S (2007). Histological study of blastema tissue genesis processes, autografted in New Zealand rabbit derm. Med Uni Arak J.

[18] Baghaban Eslaminejad M, Bordbar S (2012). Blastema from rabbit ear contains progenitor cells comparable to marrow derived mesenchymal stem cells. Vet Res Forum.

[19] Baghaban Eslaminejad M, Bordbar S (2013). Isolation and characterization of the progenitor cells from the blastema tissue formed at experimentally-created rabbit ear hole. Iran J Basic Med Sci.

[20] Mahmoudi Z, Moghaddam-Matin M, Saeinasab M, Nakhaei-Rad S, Mirahmadi M, Mahdavi-Shahri N (2011). Blastema cells derived from rabbit ear show stem cell characteristics. J Cell Mol Res.

[21] Javanmard A, Bahrami AR, Mahmoudi Z, Saeinasab M, Mahdavi-Shahri N, Matin MM (2016). Studying the expression patterns of OCT4 and SOX2 proteins in regenerating rabbit ear tissue. World Rabbit Sci.

[22] Saeinasab M, Matin MM, Rassouli FB, Bahrami AR (2016). Blastema cells derived from New Zealand White rabbit’s pinna carry stemness properties as shown by differentiation into insulin producing, neural, and osteogenic lineages representing three embryonic germ layers. Cytotechnology.

[23] Masaki H, Ide H (2007). Regeneration potency of mouse limbs. Dev Growth Differ.

[24] Behfar M, Sarrafzadeh-Rezaei F, Hobbenaghi R, Delirezh N, Dalir-Naghadeh B (2011). Adipose derived stromal vascular fraction improves early tendon healing: an experimental study in rabbits. Vet Res Forum.

[25] Javanmardi S, Sarrafzadeh-Rezaei F, Hobbenaghi R, Dalir-Naghadeh B (2008). Use of undifferentiated cultured bone marrow-derived mesenchymal stem cells for DDF tendon injuries repair in rabbits: a quantitative and qualitative histopathological study. Iran J Vet Surg.

[26] Woessner JF (1961). The determination of hydroxyproline in tissue and protein samples containing small proportions of this amino acid. Arch Biochem Biophys.

[27] Gaspar D, Spanoudes K, Holladay C, Pandit A, Zeugolis D (2015). Progress in cell-based therapies for tendon repair. Adv Drug Deliv Rev.

[28] Smith RK, Korda M, Blunn GW, Goodship AE (2003). Isolation and implantation of autologous equine mesenchymal stem cells from bone marrow into the superficial digital flexor tendon as a potential novel treatment. Equine vet J.

[29] Kosaka M, Nakase J, Hayashi K, Tsuchiya H (2016). Adipose-derived regenerative cells promote tendon-bone healing in a rabbit model. Arthroscopy.

[30] Daher RJ, Chahine NO, Razzano P, Patwa SA, Sgaglione NJ, Grande DA (2011). Tendon repair augmented with a novel circulating stem cell population. Int J Clin Exp Med.

[31] Azad-Tirgan M, Sarrafzadeh-Rezaei F, Malekinejad H, Hobbenaghi R, Heshmatian B (2016). Evaluation of tendon healing using fibroblast like synoviocytes in rabbits: a biomechanical study. Vet Res Forum.

[32] Uysal AC, Mizuno H (2010). Tendon regeneration and repair with adipose derived stem cells. Curr Stem Cell Res Ther.

[33] Behfar M, Javanmardi S, Sarrafzadeh-Rezaei F (2014). Comparative Study on functional effects of allotransplantation of bone marrow stromal cells and adipose derived stromal vascular fraction on tendon repair: a biomechanical study in rabbits. Cell J.

[34] Yeung CK, Guo X, Ng YF (2006). Pulsed ultrasound treatment accelerates the repair of Achilles tendon rupture in rats. J Orthop Res.

[35] Witvrouw E, Mahieu N, Roosen P, McNair P (2007). The role of stretching in tendon injuries. Br J Sports Med.

[36] Constantinos N, Maganaris CN, Narici MV, Maffulli N, Renström P, Leadbetter WB (2005). Mechanical properties of tendons. Tendon injuries: Basic Science and Clinical Medicine.

[37] Dowling B, Dart A (2005). Mechanical and functional properties of the equine superficial digital flexor tendon. Vet J.

[38] Bonifasi-Lista C, Lakez SP, Small MS, Weiss JA (2005). Viscoelastic properties of the human medial collateral ligament under longitudinal, transverse and shear loading. J Orthop Res.

[39] Wang JH (2006). Mechanobiology of tendon. J Biomech.

[40] Mow VC, Huiskes R ( 2005). Basic orthopaedic biomechanics and mechanobiology.

[41] Oryan A, Moshiri A (2011). A long term study on the role of exogenous human recombinant basic fibroblast growth factor on the superficial digital flexor tendon healing in rabbits. J Musculoskelet Neuronal Interact.

[42] Sharma P, Maffulli N (2005). Tendon injury and tendinopathy: healing and repair. J Bone Joint Surg.

[43] González-Quevedo D, Martinez-Medina I, Campos A, Campos F, Carriel V (2018). Tissue engineering strategies for the treatment of tendon injuries: a systematic review and meta-analysis of animal models. Bone Joint Res.

[44] Lin Y, Chen X, Yan Z, Liu L, Tang W, Zheng X (2006). Multilineage differentiation of adipose-derived stromal cells from GFP transgenic mice. Mol Cell Biochem.

[45] Gong F, Cui L, Zhang X, Zhan X, Gong X, Wen Y (2018). Piperine ameliorates collagenase-induced Achilles tendon injury in the rat. Connect Tissue Res.

[46] Naderi S, Khayat Zadeh J, Mahdavi Shahri N, Nejad Shahrokh Abady K, Cheravi M, Baharara J (2013). Three-dimensional scaffold from decellularized human gingiva for cell cultures: glycoconjugates and cell behavior. Cell J.

[47] Mahdavi Shahri N, Akbarzadeh Niaki M, Moghadam Matin M, Fereidoni M, Lari R (2014). The histological study of the interactions between rabbit decellularized esophagus scaffold and the blastema tissue obtained from the pinna of New Zealand White rabbit. J Isfahan Med Sch.

[48] Dahmardeh T, Mahdavi Shahri N, Moghadam Matin M, Behnam Rassouli M, Lari R (2016). Inducing the effects of acellular dermal matrix on blastema tissue originated from the pinna of New Zealand White rabbit, in vitro. Gene Cell Tissue.

[49] Baharara J, Mahdavishahri N, Saghiri N, Rasti H (2012). Histological study of interaction between blastema tissue and decellularized three-dimensional matrix of bladder. Zahedan J Res Med Sci.

